# Shared Principles of Individuation Underpin Object–Event Correspondences Cross‐Linguistically

**DOI:** 10.1111/cogs.70248

**Published:** 2026-08-19

**Authors:** Sarah Hye‐yeon Lee, Anna Papafragou

**Affiliations:** ^1^ Department of Linguistics University of Pennsylvania

**Keywords:** Object, Event, Individuation, Event cognition, Telicity, Count–mass

## Abstract

Entities in both the spatial and the temporal domains are characterized by a shared notion of individuation. Within the spatial domain, *objects* (e.g., table, ball) are discrete, countable mental individuals, and are distinct from unindividuated *substances* (e.g., sand, water). Within the temporal domain, *bounded events* (e.g., pile up a deck of cards) are mental individuals: They are internally structured in terms of distinct temporal stages and a well‐defined endpoint; as such, they differ from *unbounded events* (e.g., shuffle a deck of cards) that lack such features. A key question left open by previous studies (conducted with English speakers) is whether the principles of mental individuation across the spatial and temporal domains are universal or shaped by one's native language. Here, we address this issue by comparing English and Mandarin speakers. Our results highlight shared signatures of individuation that underpin object–event correspondences across languages and are potentially universal.

## 1 Introduction

### 1.1 Objects and events as mental individuals

Humans understand, interact with, and talk about the world by organizing their continuous experience of space and time into different types of mental units. In the spatial domain, *objects* serve as the basic units for many human perceptual and cognitive processes from infancy to adulthood (Carey & Xu, 2001; Piaget, 1955; Quine, 1960; Scholl, 2001; Spelke, 1988, 1994). In the temporal domain, *events* are the foundational units for human perception and cognition across the lifespan (Baldwin, Baird, Saylor, & Clark, 2001; Hespos, Ferry, & Rips, 2009; Sharon & Wynn, 1998; Zacks & Tversky, 2001). The focus of the current study is to investigate the relationship between these mental units and language. Specifically, we are interested in whether the principles that support mental units within and across the spatial and temporal domains are shared across speakers of different languages.

Closer inspection reveals more specific subdivisions as well as important commonalities in the mental entities across the spatial and temporal domains. Within the spatial domain, there is an important conceptual distinction between *objects* (e.g., table, ball) and *substances* (e.g., sand, water; see Prasada, Ferenz, & Haskell, 2002), a distinction that originates in Aristotle's (Aristotle, 350 B.C.E./1994) analysis of form and matter. Objects are understood to possess form, whereas substances are conceived to lack an inherent form and are themselves matter. It has been argued that an object construal, unlike a substance construal, involves conceiving an entity's structure as non‐arbitrary (Prasada et al., 2002). For example, when we think of an entity as a table, we understand it to have a particular structure because it is a table. On the other hand, some wood can be in any arbitrary configuration. This conceptual distinction influences the privileged status of objects in entity tracking and quantification among both adults and children (e.g., Chiang & Wynn, 2000; Hespos et al., 2009; Huntley‐Fenner, Carey, & Solimando, 2002; Soja, Carey, & Spelke, 1991; van Marle & Scholl, 2003; van Marle & Wynn, 2011).

Similarly, within the temporal domain, there is a conceptual distinction between *bounded* events (sometimes simply referred to as *events*) that are understood to have distinct temporal stages and a well‐defined endpoint (e.g., put up one's hair) and *unbounded* events that lack these features (e.g., scratch one's hair; see Ji & Papafragou, 2020a, 2020b, 2022, 2024). For example, an event of putting up one's hair progresses toward a natural endpoint—a point at which the hair is fully put up. In contrast, an unbounded event like scratching one's hair lacks such an inherent endpoint. The event can terminate at any arbitrary moment. Although this distinction has not been studied as much as the object–substance distinction, there is growing evidence that boundedness is a feature that is processed and computed during online event apprehension (Ji & Papafragou, 2022; Vurgun, Ji, & Papafragou, 2024).

Before we proceed, we note that both the object–substance distinction in the spatial domain and the boundedness–unboundedness distinction in the temporal domain are issues of *construal* in the human mind. The physical world itself does not a priori provide these distinctions. In fact, one can conceive of the same physical entity as either a sandcastle (object) or some sand (substance). Similarly, the same happening in the world can be understood as either running to the store (bounded event) or running (unbounded event).

More relevant for present purposes, as pointed out recently by Papafragou and Ji (2023), there are similarities in how events and objects are represented as cognitive entities. In that study, after brief training, viewers were able to extend categories of bounded or unbounded events to objects or substances, respectively. For example, they were able to extend a category consisting of bounded events (e.g., dress a teddy bear) to also include novel objects (e.g., a solid, ring‐like entity) and a category consisting of unbounded events (e.g., pat a teddy bear) to also include novel substances (e.g., a white non‐solid mass). Viewers were able to draw such connections between events and objects even in the absence of prior training. Furthermore, when the experimenters tried to teach participants to draw unnatural generalizations between the two domains (e.g., by generalizing bounded events to substances or unbounded events to objects), participants failed to learn them and, in fact, reverted to the natural, *reverse* generalizations at test.

Here, we take the perspective that the strong mental alignment between bounded/unbounded events and objects/substances is supported by a shared notion of individuation that runs across domains (Lee, Ji, & Papafragou, 2024; Papafragou & Ji, 2023; for relevant perspectives, see also Bach, 1986; Casati & Varzi, 2008; Kuhn, Geraci, Schlenker, & Strickland, 2021; Wellwood, Hespos, & Rips, 2018). On this account, developed in detail in Lee et al. (2024), objects and bounded events both qualify as individuals because they are understood to possess a well‐defined internal structure; by contrast, substances and unbounded events are non‐individuals because they are understood to lack such an inherently well‐defined structure. On this account, a well‐defined structure is one in which internal parts are organized in a predetermined manner. For example, parts of a table such as the table legs and the tabletop are arranged in a well‐defined spatial configuration: A table leg does not sit above the tabletop. Similarly, the stages (parts) of putting up one's hair must proceed in a pre‐defined temporal order: Gathering the hair can never precede putting a hairpin in.

Such a structure gives rise to a set of principles that apply across objects and bounded events (Lee et al., 2024). According to the first of these principles known as “No Restructuring,” individuated entities across domains resist restructuring because they are understood to be organized with a specific (spatial or temporal) structure that cannot be rearranged. In the spatial domain, this means that the spatial parts of individual objects (e.g., the tabletop and the table legs of a table) are organized in a designated manner and cannot be rearranged, unlike different parts of a substance (e.g., grains in sand) that are not subject to such a restriction. In the temporal domain, the No Restructuring principle means that bounded events proceed in a way in which the parts are temporally arranged in a well‐defined manner, which cannot arbitrarily be rearranged. However, parts of unbounded events (e.g., the third and fifth steps in a walking event) can be switched or rearranged without altering the nature of the event. In an experiment designed to test the No Restructuring principle, when an image of an object (e.g., a vase) was edited to depict changes in structure, viewers were more likely to notice the change than when an image of a substance (e.g., some clay) was edited in the same way. Similarly, when a video depicting a bounded event (e.g., folding a handkerchief) was edited with changes in structure, viewers were more likely to notice the change than when a video depicting an unbounded event (e.g., waving a handkerchief) was edited in the same manner (Lee et al., 2024).

According to a second principle of individuation known as the “Distinct Parts” principle, individuated entities alone are understood to have distinct parts (Lee et al., 2024). That is, two random subparts of an object (such as a tabletop and a table leg of a table) are more likely to be considered distinct than two random subparts of a substance (two portions of sand). Similarly, two random subparts of a bounded event (e.g., folding a handkerchief) are more distinct than two random subparts of an unbounded event (e.g., waving a handkerchief). In Lee et al. (2024), viewers perceived subparts of individuated entities (objects and bounded events) as more distinct from one another than subparts of non‐individuated entities (substances and unbounded events).^1^ Taken together, these principles support the conceptual distinction between mental individuals and nonindividuals and reveal connections among the basic mental units across the spatial and temporal domains.

### 1.2 Cross‐linguistic differences and individuation

A key issue left open by previous studies is whether the principles for mental individuation across the spatial and temporal domains vary across speakers of different languages. The studies summarized above—including the object–event correspondence studies in Papafragou and Ji (2023) and Lee et al. (2024)—have only tested English speakers.

This issue is important because languages vary in the ways they refer to and quantify different types of entities. In many languages, there is a grammatical distinction between count and mass nouns, which is reflected in the syntactic behavior of these nouns. In English, for example, count nouns like *vase* can be used in either their singular (e.g., *This is a vase*) or plural forms (e.g., *These are vases*) but not in their bare form (e.g., *This is vase). On the other hand, mass nouns such as *sand* are used in their bare form (e.g., *This is sand*) but not in their singular or plural forms (e.g., *This is a sand/*These are sands).^2^ Count nouns can directly combine with numeric quantifiers (e.g., *three vases*) but mass nouns cannot (e.g., *three sands). In these languages, count syntax often provides a cue to individuation (Bloom, 1999; Gordon, 1985; Link, 1983). Speakers of English know that *a table* refers to a discrete individuated entity as opposed to some arbitrary portion of a table. It has also been shown that count–mass syntax affects individuation in quantity judgment tasks. In Barner and Snedeker's (2005) study, quantity judgments were based on number when words were presented in count syntax (e.g., “Who has more stones?”), whereas judgments were based on mass/volume when the same words were presented in mass syntax (e.g., “Who has more stone?”).

Other languages, however, do not make the syntactic distinction between count and mass nouns. For instance, Mandarin Chinese lacks count–mass syntax, thus all nouns can appear in their bare form (Chierchia, 1998). In a Mandarin sentence such as “Zuótiān wǒ mǎi le shū ‘Yesterday, I buy ASP book(s)’,” the bare noun *shū* “book” can either denote a single book or plural books (Rullman & You, 2006, p. 175). When quantifying nouns, Mandarin uses classifiers both with nouns that refer to individuals (e.g., *sān běn shū* “three CL book”) and with nouns that do not (e.g., *sān píng jiǔ* “three CL wine”). Based on this property, some authors have claimed that all nouns in classifier languages are syntactically mass nouns (Allan, 1980; Chierchia, 1998, 2010; Krifka, 1995) and that Mandarin nouns do not connect to the distinction between objects and substances (Lucy, 1996). Opponents of this view have suggested that, instead, the lexical semantics of nouns (Cheng & Sybesma, 1998, 1999; Zhang, 2013), together with classifier type (Borer, 2005; Cheng & Sybesma, 1998, 1999; Pelletier, 2012), may provide criteria of individuation for Mandarin. Experimental evidence so far supports the latter view (Cheung, Li, & Barner, 2012; Zhu & Syrett, 2022). On both views, however, it is clear that Mandarin does not grammatically mark the distinction between individuated and unindividuated entities in the same way that mass–count languages like English do.

Turning to the verbal domain*, telicity* is a notion that refers to the internal temporal structure of events (Beavers, 2008; Dowty, 1979; Hay, Kennedy, & Levin, 1999; Jackendoff, 1996; Kennedy & Levin, 2008; van Hout, 1996; Vendler, 1957; Verkuyl, 1993; see Filip, 2012, for an overview) and is related to the conceptual notion of boundedness (Ji & Papafragou, 2020a, 2022). Telic predicates (e.g., *build a sandcastle*) describe events that develop toward a natural, inherent endpoint, whereas atelic predicates (e.g., *play with sand*) describe events that lack such an endpoint. In telic predicates, once the endpoint is reached, the event is said to have *culminated*. While telicity refers to the inherent temporal structure of the event, culmination concerns whether the inherent endpoint has been reached. The two notions are closely related because atelic predicates lack a culmination point. Therefore, it is specifically events described by telic predicates that can culminate.^3^

In English, telic predicates (e.g., accomplishment predicates that semantically encode both an activity and an endpoint) typically entail event culmination when they are in non‐progressive aspect (e.g., Vendler, 1957). For example, in English, *I fixed the car* entails that fixing is completed, and the car is fixed. This entailment cannot be canceled, so it would be infelicitous to say *I fixed the car but I didn't finish fixing it*. This pattern, however, is not universal across languages. It has been found that in many other languages, accomplishment predicates do not entail event culmination, unless some other linguistic material specifies it. Mandarin is one such language where the counterparts of English accomplishment verbs can give rise to non‐culminating readings (Klein, Li, & Hendriks, 2000; Smith,^1990^, ^1997^; Soh & Kuo, 2005; Sybesma, 1997, 1999; Tai, 1984; see also Filip, 2001, 2008; Rothstein, 2004, for Russian; Kratzer, 2004, for Finnish; Singh, 1991, for Hindi; Kardos, 2016, for Hungarian). Based on these observations, some have argued that all monomorphemic accomplishment verbs in Mandarin are atelic, with a few exceptions (Lin, 2004; Ross, 1995; Tai, 1984). Instead, Mandarin resultative verb compounds (e.g., *da‐sui* “hit‐smash”), in which the second verb (*sui* “smash”) explicitly encodes the resultant state that is associated with the event described by the first verb (*da* “hit”), are used as telic predicates.^4^

Importantly, telicity is not a property of the verb alone but is determined compositionally—arising from the interaction between the verb and its nominal argument(s). For instance, in English, the predicate *eat an apple* is telic because the quantized noun phrase *an apple* contributes the endpoint of the event. In contrast, mass nouns or bare plurals, as in *eat apples* or *eat jello* yield atelic readings. Quantized arguments “measure out” the event incrementally as they undergo change of state, with the specific quantity of the object delimiting the event (e.g., Beavers, 2012; Dowty, 1977, 1979; Krifka, 1989, 1992, 1998; Rothstein, 2004; Tenny, 1987; Vendler, 1957; Verkuyl, 1972, 1993). However, as noted earlier, Mandarin bare noun phrases do not provide any information about the quantity of the referent, as the language lacks an overt count/mass and singular/plural distinction. As a result, bare noun phrases in Mandarin typically do not contribute to a telic interpretation of the verb phrase.^5^ This means that in Mandarin, a verb phrase that corresponds to “eat apple” can either describe an event that has an inherent endpoint (eating a specific quantity of apples) or not (eating an unspecified quantity of apples). Therefore, while both English and Mandarin speakers can both express the telic‐atelic distinction, they differ in the way they do so, as well as in the extent to which it is grammatically obligatory.

Could these cross‐linguistic differences in the nominal and verbal domains affect non‐linguistic individuation? One possibility is that the principles linked to the structure of mental individuals (objects and bounded events; Lee et al., 2024) arise in a viewer's mind through language (count syntax and telicity of predicates; see Quine, 1960). If so, we cannot rule out the idea that the findings pointing to object–event correspondences in cognition are directly related to the fact that speakers of English obligatorily use such grammatical devices to express notions related to individuation and might not extend (or extend less clearly) to Mandarin speakers. Another possibility is that the conceptual principles of individuation are universally shared, regardless of the way individuation is expressed in one's language. This would be compatible with the view that non‐verbal concepts of individuation support relevant linguistic categories (Soja, Carey, & Spelke, 1992).

In the nominal domain, some researchers have argued in favor of the first possibility, offering experimental evidence that speakers of classifier languages such as Japanese or Yucatec Maya conceptualize the object–substance distinction differently from speakers of mass–count languages such as English (Imai & Gentner, 1997; Lucy, 1992). Later work, however, has cast doubt on this proposal by showing that these differences emerge when participants covertly use lexical items in their native language to categorize novel spatial entities: When participants are asked to categorize objects and substances without access to linguistic labels, and presumably have to rely on non‐linguistic individuation criteria, the cross‐linguistic differences in entity construal disappear (Li, Dunham, & Carey, 2009; cf., also Barner, Inagaki, & Li, 2009; Imai & Mazuka, 2003; Papafragou, 2017). This debate could extend to event individuation since the logical hypothesis space is similar, but relevant data are sparse. Moreover, we currently lack data on whether a single set of conceptual signatures of individuation generalizes to *both* objecthood and eventhood in similar ways across speakers of different languages. Importantly, this question cannot be settled by research on mechanisms of individuation within the spatial and temporal domains separately because such research leaves open different possibilities about how these mechanisms operate: For instance, one salient possibility is that the fundamental conceptual considerations relevant to the object–substance distinction are specific to the spatial domain and are unrelated to the bounded–unbounded event distinction. An alternative possibility is that signatures of individuation are domain‐general, supporting both objecthood and eventhood (Lee et al., 2024). In what follows, we test these alternative hypotheses directly.

Specifically, in the current study, we investigate whether the distinction between individuated and unindividuated entities across the object and event domains is conceptualized in similar ways by speakers of English and Mandarin Chinese. We use the paradigm of Lee et al. (2024) and focus on the principles of No Restructuring and Distinct Parts that have been argued to characterize both spatial and temporal individuals. If conceptual individuation across objects and events is a product of the human mind prior to (and independently from) language, speakers of English and Mandarin should both abide by these principles in similar ways, regardless of domain (just as in the original findings from Lee and colleagues). Alternatively, construals of individuation could diverge cross‐linguistically: In that case, English and Mandarin speakers might conceptualize individuals (objects and bounded events) differently in their respective (spatial and temporal) domains, such that the principles that support cross‐domain correspondences would be distinct across different language groups. We test these competing predictions in Experiments 1 (No Restructuring principle; 1a: Objects, 1b: Events) and 2 (Distinct Parts principle; 2a: Objects, 2b, 2c: Events).

Results from this study are important for two reasons. First, they can throw light onto the source and nature of the object–event correspondence and inform the fundamental mechanisms that enable humans to parse everyday experiences into units across the spatial and temporal domains. Second, they can contribute to broader efforts to understand how language connects to human cognition (for different perspectives on this topic, see Choi & Bowerman, 1991; Gleitman & Papafragou, 2005; Landau, 2022; also reviews in Bowerman & Levinson, 2001; Gentner & Goldin‐Meadow, 2003; Wolf & Holmes, 2011).

## 2 General methods

### 2.1 Transparency and openness

This research was conducted under the approval of the institutional review board at the University of Pennsylvania. Informed consent was obtained from all participants. All stimulus materials, trial‐level data, and analysis code are available on Open Science Framework via this link: https://osf.io/k487a. Analyses were conducted using the *glmmTMB* package (Brooks et al., 2017) in the R software environment (R Core Team, 2024). In addition, we conducted supplemental Bayesian analyses using the *brms* package (Bürkner, 2017). The experiments were not preregistered.

### 2.2 General participant recruitment and profile

Throughout these experiments, we recruited adult native speakers of English and native speakers of Mandarin Chinese from the online recruitment platform Prolific. Participants were prescreened using the built‐in language‐related screeners in Prolific, such that the study was only visible to participants who identified English and Mandarin as their first language, primary language, and fluent language. In addition, for the Chinese‐speaking group, we conducted an informal native speaker check with five advanced‐level Mandarin questions (e.g., double‐embedded relative clauses, advanced‐level vocabulary, word order involving spatial adverbials). Participants in our experiments scored 95.10% on average, on par with the scores obtained from a separate group of speakers (*N *= 21) recruited from the Beijing Institute of Technology in Beijing (95.24%). According to self‐reported data, the recruited Mandarin speakers generally spoke Mandarin from birth as native speakers, were almost all born in a Mandarin‐speaking environment, and, on average, lived in such an environment until adulthood (at the time of testing, they resided in various parts of the world including North America, Europe, Australia, and Asia). As is commonly the case, Mandarin‐speaking participants also spoke English (average self‐reported proficiency in English was 5.73 out of 10). As we later show, neither length of residence abroad nor proficiency in English affected this group's performance in our experiments (see Section 4.3.4).

## 3 Experiment 1: No Restructuring principle

According to the No Restructuring principle, individuated entities resist restructuring (Lee et al., 2024). This principle predicts that observers would find restructurings to individuated entities (objects, bounded events) more noticeable than those to unindividuated entities (substances, unbounded events). If cross‐domain individuation is shared across speakers of different languages, both English and Mandarin‐speaking adults should show sensitivity to the No Restructuring principle for both spatial and temporal entities. But if the conceptualizations of individuals are shaped by linguistic encoding of count syntax or telicity of predicates, English speakers should be exclusively, or more strongly, sensitive to the No Restructuring principle. In Experiment 1, we test these predictions with adult English and Mandarin speakers, using the experimental stimuli and paradigm from Lee et al. (2024).

### 3.1 Experiment 1a: Objects

#### 3.1.1 Participants

Forty‐eight adult native speakers of English and 47 adult native speakers of Mandarin Chinese were recruited. One additional Mandarin speaker participated but was excluded from data analysis due to data error. All participants in this and the following experiments were paid at an 8 USD/h rate. The sample size was determined on the basis of a pilot study with 10 English‐speaking participants reported in Lee et al. (2024) using the same design: That study indicated that a sample size of at least 21 was needed to detect an effect of condition within a single language with 90% power at significance level *α* = 0.01 (pilot Cohen's *d* = 0.909; Lee et al., 2024, used 25 participants per language).

#### 3.1.2 Stimuli

Following Lee et al. (2024), the target stimuli consisted of 16 images of familiar objects (e.g., crystal swan) and 16 images of the corresponding substances (e.g., crystal; see Table 1). Each object (e.g., crystal swan) was paired with the corresponding substance (e.g., crystal) to create 16 object–substance pairs, for a within‐subject design where participants saw either an object or a substance from a given pair but never both. As in Lee et al. (2024), we created spatially restructured versions of each target entity by switching the positions of the second and third vertical strips of the image (see Table 2). Images were edited using the Adobe Photoshop 2022 software. All images were in 400 × 400 pixel dimensions.

**Table 1:** List of image stimuli in Experiments 1a and 2a

	Object	Substance
1	Onigiri	Rice
2	Concrete block	Cement powder
3	Gold bracelet	Gold nuggets
4	T‐shirt	Cotton wool
5	Ball of yarn	Yarn threads
6	Vase	Clay
7	Tomato	Chopped tomatoes
8	Key	Metal
9	Wooden bowl	Chopped wood
10	Onion	Chopped onions
11	Roll of toilet paper	Toilet paper
12	Meatball	Ground meat
13	Crystal swan	Crystal
14	Sandcastle	Sand
15	Pot	Terra cotta clay
16	Lotion (bottle)	Lotion (material)

**Table 2:** Sample images in Experiment 1a

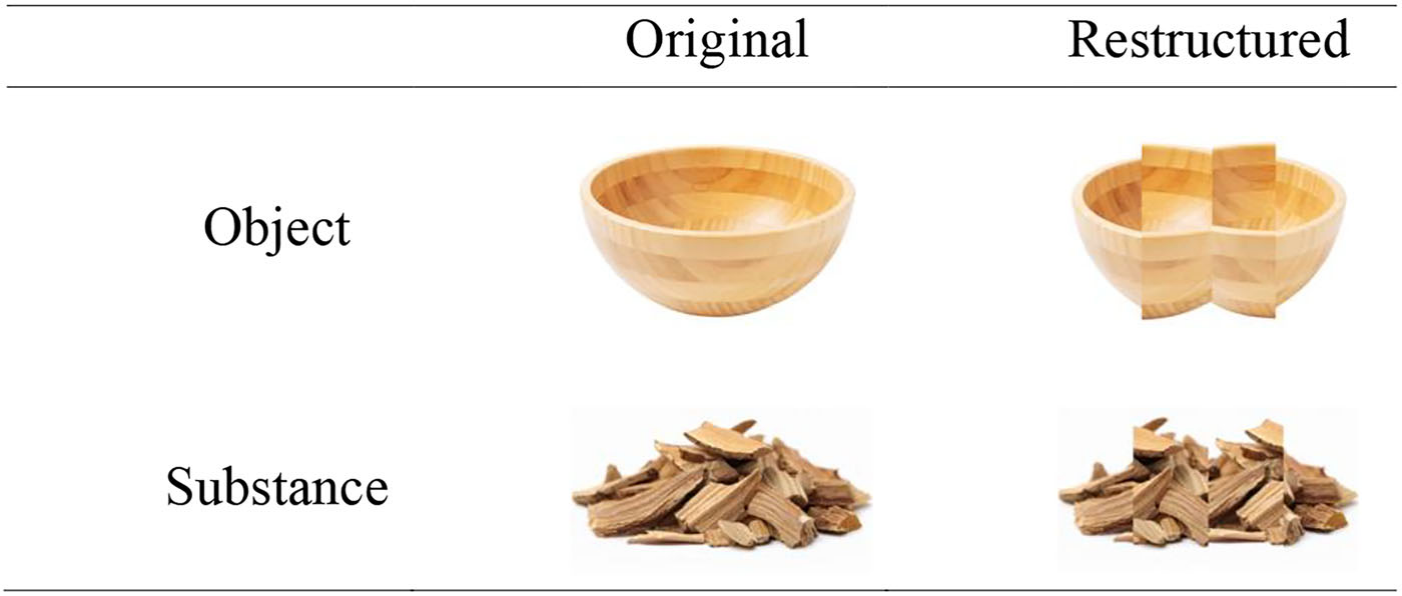

*Note*. Images in this table are representative of the stimuli used in the experiment but are not the actual stimuli due to copyright restrictions. The actual stimuli can be accessed in full on our OSF page.

The original stimuli in Lee et al. (2024) came from a pool of images that were normed by asking 15 naïve native English speakers to rate the entities in their original (not restructured) form on a scale of 1–7, with 1 being a “*good object*” and 7 being a “*good substance*” (see Li et al., 2009). Items categorized as objects had a mean rating of 2.62 (*SD* = 2.25), and items categorized as substances had a mean rating of 4.81 (*SD* = 2.31), with people rating substances higher than objects on our response scale (*t*(14) = −7.1, *p* < .001). Such items are known to elicit strong English–Mandarin differences in how they are encoded (Li et al., 2009), such that English speakers typically encode object items with count nouns and substance items with mass nouns (see also Prasada et al., 2002), whereas Mandarin does not grammatically distinguish the two types of items.

We also included a set of 12 filler items, six of which were objects (e.g., spatula) and the other six substances (e.g., juice). For half of them, we also created versions of the images that differed only subtly in color.

#### 3.1.3 Procedure

All experiments reported in this study were hosted online on PennController IBEX (Zehr & Schwarz, 2018; https://www.pcibex.net/), and participants completed them remotely via the Internet. In all experiments, all recruitment materials and instructions were provided in the participants’ native language.

At the beginning of each target trial, a fixation cross was displayed for 1000 ms. After the fixation cross, the original (not restructured) image was displayed for 100 ms. Then, the screen was masked with a gray mask for 3000 ms. Afterward, the restructured image was displayed for 100 ms. There was no post‐mask after the restructured image. Participants were asked to indicate whether they thought the two images were identical, by using a button (English: “Edited (E) Identical (I)”; Mandarin: “剪辑过 (F) 还是相同的 (J)”). The display advanced after the button press.

Two presentation lists were created using a standard Latin Square design. Participants were randomly assigned to one of the two lists. Each list contained 16 target items, which consisted of eight objects and eight substances. Entity type (Object, Substance) was a within‐subject condition, and therefore participants saw either an object or a substance from each object–substance pair but never both. Both lists also contained the same set of 12 filler items. In six of the filler trials, participants saw identical images before and after the 3000 ms mask. In the other six of the filler trials, the images that participants saw before and after the mask differed subtly in color. This ensured that there was a variety of yes and no correct responses throughout. Target and filler items were pseudo‐randomly distributed throughout the list, and this presentation order was identical across lists, for all participants. The same approach to experimental design was used in all experiments.

#### 3.1.4 Results and discussion

In all experiments reported in this study, the accuracy of the participants’ responses was analyzed using generalized linear mixed effects models. In addition to the frequentist analyses reported in the paper, we conducted supplemental Bayesian analyses for all experiments to address potential concerns about a Type II error for the interaction effects. We report full results in the Supporting Information. Results of the Bayesian analyses indicated the same set of findings as our frequentist analyses.

Models were estimated using the *glmmTMB* package (Brooks et al., 2017) in R (R Core Team, 2024). Whenever possible, we report results from models with maximal random effects structure. When the maximal model failed to converge, the random effects structure was reduced, starting with by‐item effects, until the model converged. For all experiments, we note any convergence issues and present the next model that converged following the approach above.

**Fig. 1:**
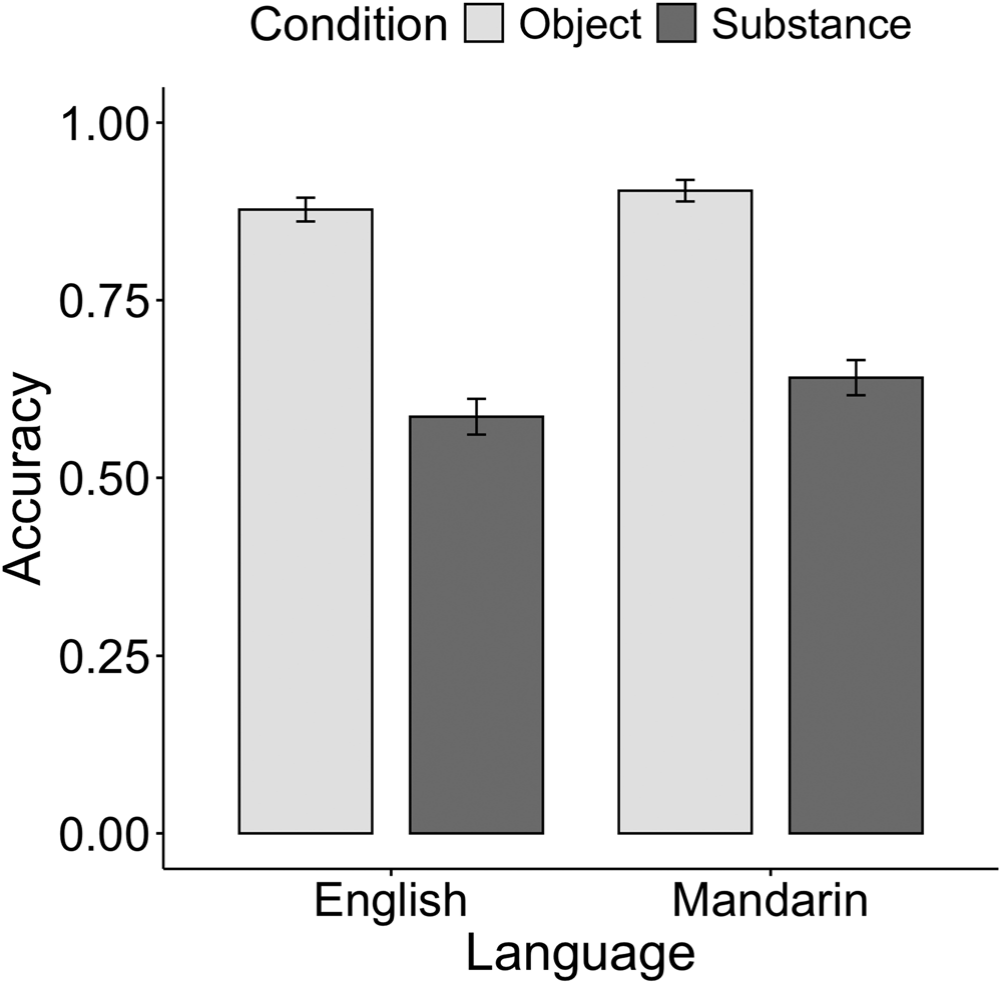
Mean accuracy by Language and Condition in Experiment 1a. (Error bars represent ± *SE*.)

Results from Experiment 1a are shown in Fig. 1. We coded Condition (Object = 0.5, Substance = −0.5) and Language (English = 0.5, Mandarin = −0.5) using centered contrasts and included them as fixed effects. As random effects, we entered intercepts for participants and items, as well as by‐participant random slopes for Condition and by‐item random slopes for Condition and Language, and their interaction (the model with the maximal random effects structure did not converge). We found an effect of Condition, such that participants were more likely to accurately judge that the original and the restructured images were different when presented with Objects than with Substances (*β* = 2.62, *SE* = 0.39, *z *= 6.65, *p* < .001). There was no effect of Language (*β* = −0.26, *SE* = 0.30, *z* = −0.86, *p* = .392), nor a Condition by Language interaction (*β* = 0.06, *SE* = 0.48, *z* = 0.13, *p* = .899). Both groups of participants were better at detecting restructurings to Objects than to Substances.

These results support the No Restructuring principle. Recall that, according to this principle, individuals, such as objects, possess a well‐defined structure that resists change. Both English and Mandarin speakers were sensitive to this principle in the object domain.

### 3.2 Experiment 1b: Events

#### 3.2.1 Participants

Forty‐seven new adult native speakers of English and 46 new adult native speakers of Mandarin Chinese participated. One additional English speaker participated but did not finish the experiment.

#### 3.2.2 Stimuli

For target items, we used 16 bounded and 16 corresponding unbounded videos from Lee et al. (2024; see Table 3). Each bounded event (e.g., dress a teddy bear) was paired with the corresponding unbounded event (e.g., pat a teddy bear) to create 16 bounded‐unbounded event pairs, for a within‐subjects design. These were drawn from a larger set of stimuli used in Ji and Papafragou (2020a). Corresponding bounded and unbounded videos were matched for duration (duration range: 4.5–13 s; *M* = 7.98 s). All videos involved the same girl doing a familiar everyday action in a lab room. The contrast between bounded and unbounded events was derived from two sources. In half of the stimuli (Items 1–8 in Table 1), the nature of the action differed across the pair. The bounded event video involved an action that caused a clear change‐of‐state in the object (e.g., dress a teddy bear), but the unbounded one did not depict a change‐of‐state (e.g., pat a teddy bear). In the other half of the stimuli (Items 9–16 in Table 1), the bounded and unbounded videos depicted the same action but differed in terms of the nature of the affected object. The bounded event video depicted action on a single individual (e.g., eat a pretzel), while the unbounded counterpart showed the same action on either an unspecified plurality of objects or a mass quantity (e.g., eat Cheerios).

**Table 3:** List of video stimuli in Experiments 1b and 2b

No.	Bounded Events	Unbounded Events
1	Scoop up yogurt	Stir yogurt
2	Fold a handkerchief	Wave a handkerchief
3	Stack a deck of cards	Shuffle a deck of cards
4	Group pawns based on color	Mix pawns of two colors
5	Roll up a towel	Twist a towel
6	Dress a teddy bear	Pat a teddy bear
7	Put up one's hair	Scratch one's hair
8	Close a fan	Use a fan
9	Eat a pretzel	Eat Cheerios
10	Stick a sticker	Stick stickers
11	Tie a knot	Tie knots
12	Tear up a tissue	Tear slices off tissues
13	Cut a ribbon in half	Cut pieces from a roll
14	Peel a banana	Crack peanuts
15	Flip a postcard	Flip pages
16	Blow a balloon	Blow bubbles

The videos had been normed to ensure that they would illustrate the contrast in boundedness (Ji & Papafragou, 2022): English‐speaking participants (*n* = 40) judged videos of bounded events as “something with a beginning, midpoint and specific endpoint” 87% of the time but said the same for videos of unbounded events only 21.5% of the time (a significant difference, *t*(39) = 20.33, *p* < .001). Additionally, the 20 pairs of bounded–unbounded videos that we drew our stimuli from had been used in a linguistic description task with English and Mandarin speakers (Ji & Papafragou, 2024). English speakers mostly provided telic predicates in describing bounded events (*M* = 91%), and atelic predicates in describing unbounded events (*M* = 87.3%). Mandarin speakers, however, were more likely than English speakers to use atelic descriptions for describing both bounded (*M* = 54.3%) and unbounded events (*M* = 87%). That is, English speakers and Mandarin speakers differed in how they describe bounded events. This finding confirms that there are cross‐linguistic differences in the descriptions of the video stimuli that we used.

For restructured versions of videos in Experiment 1b, we used stimuli created by Lee et al. (2024), where each original event video was temporally restructured by dividing each video into four temporal segments of equal duration and switching the second and third segments (see Table 4). This mirrors the stimuli design in Experiment 1a, where each entity was divided into four segments of equal widths, and the second and third segments were switched. Videos were edited using the Adobe Premiere Pro 2022 software.

**Table 4:** Sample videos in Experiment 1b

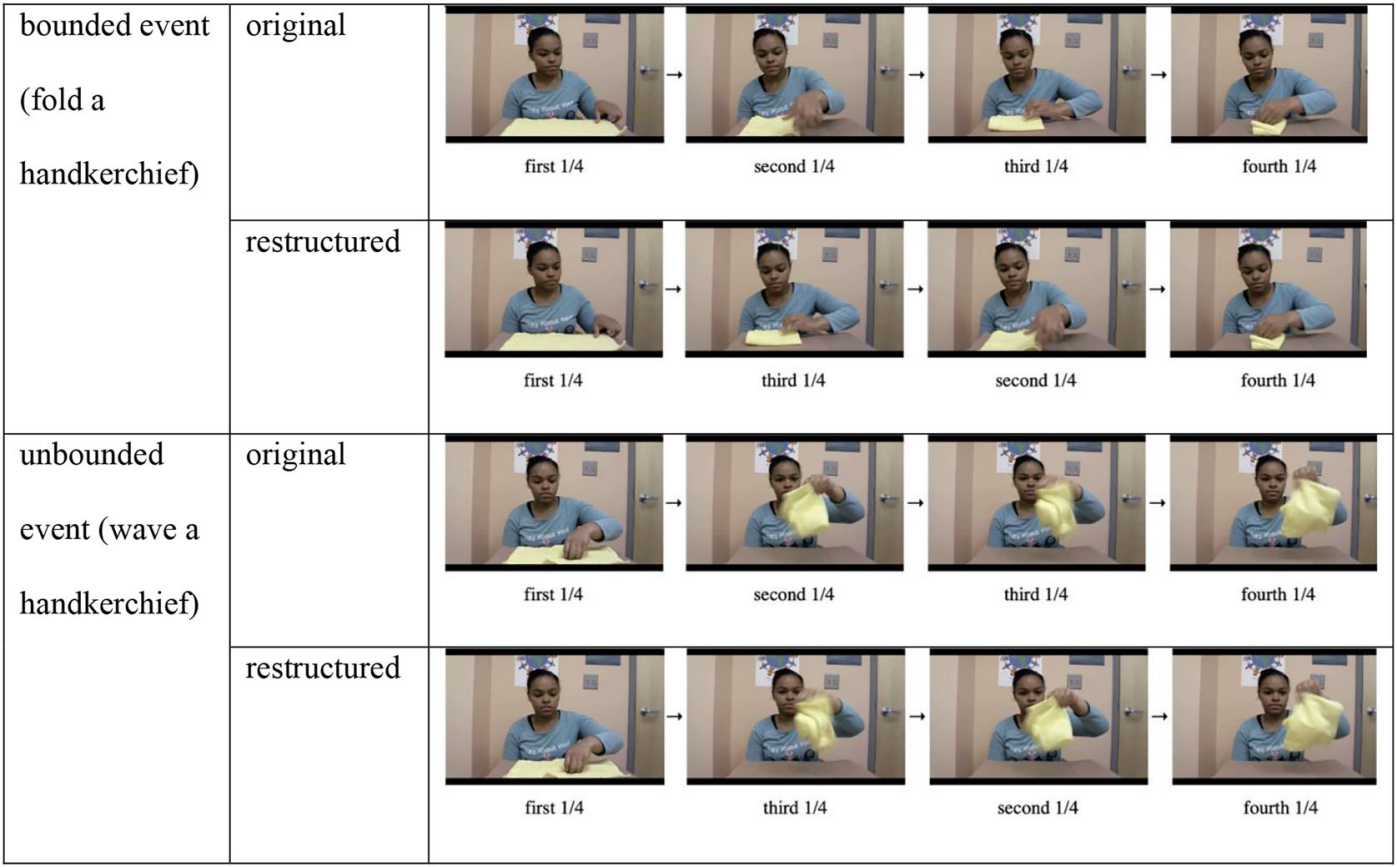

#### 3.2.3 Procedure

At the beginning of each test trial, a fixation cross was displayed for 1000 ms. Once the fixation cross disappeared, participants watched the original video. Then, the screen was masked for 1500 ms by a gray mask.^6^ Afterward, participants watched the restructured video. At the end of each trial, participants were asked to identify whether the two videos they watched were identical (English: “Edited (E) Identical (I)”; Mandarin: “剪辑过 (F) 还是相同的 (J)”).

Event type (Bounded, Unbounded) was a within‐subject condition, and therefore participants saw either a bounded or unbounded event from each bounded–unbounded event pair. Two lists were created using a standard Latin Square design. For a given target item, different conditions (Bounded, Unbounded) were included in each list. In total, each list contained eight bounded and eight unbounded events. Participants were randomly assigned to one of the two lists. In addition to these target items, participants were presented with 12 filler items. In six of the fillers, participants saw identical videos before and after the mask, and in the other six, the video shown after the mask was an edited version of the pre‐mask video. The editing involved temporally reversing segments of the video. Target and filler items were pseudo‐randomly distributed throughout the list, and this presentation order was identical for all participants, across lists.

#### 3.2.4 Results and discussion

**Fig. 2:**
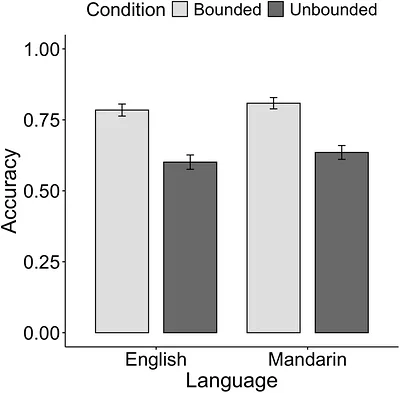
Mean accuracy by Language and Condition in Experiment 1b. (Error bars represent ± *SE*.)

Results from Experiment 1b are shown in Fig. 2. In analyzing the accuracy of participants’ responses using generalized linear mixed effects models, we included Condition (Bounded = 0.5, Unbounded = −0.5) and Language (English = 0.5, Mandarin = −0.5) as fixed effects. Random effects included intercepts for participants and items, as well as by‐participant random slopes for Condition and by‐item random slopes for Condition and Language. (The maximal model that included by‐item random slopes for the Condition × Language interaction did not converge.) There was an effect of Condition: Participants were more likely to accurately judge that the original and the restructured videos were different when presented with Bounded events than with Unbounded events (*β* = 1.40, *SE* = 0.44, *z* = 3.14, *p* < .01). There was no effect of Language (*β* = −0.29, *SE* = 0.30, *z* = −0.98, *p* = .325), nor a Condition by Language interaction (*β* = −0.10, *SE* = 0.34, *z* = −0.30, *p* = .764): Both groups of participants were better at detecting restructurings to Bounded events than to Unbounded events.

Together with results from Experiment 1a, data from Experiment 1b support the No Restructuring principle characterizing individuation (Lee et al., 2024) cross‐linguistically. As predicted by the No Restructuring principle, both English‐ and Mandarin‐speaking adults were better at detecting structural changes to individuated entities (objects, bounded events) than to non‐individuated entities (substances, unbounded events) across the spatial and temporal domains.

These results argue against the possibility that the conceptual principle of No Restructuring arises only through routine encoding of individuation in one's native language. Rather, these results support the hypothesis that conceptual individuation is a product of the human mind prior to (and independently from) the specifics of one's native language.

## 4 Experiment 2: Distinct Parts principle

The Distinct Parts principle states that individuated entities, unlike those lacking individuation, possess distinct parts (Lee et al., 2024). This principle predicts that observers would be better at detecting the difference between two random subparts of individuated entities (objects, bounded events) than the difference between two random subparts of unindividuated entities (substances, unbounded events). If conceptual signatures of individuation are broadly shared, both English‐ and Mandarin‐speaking adults should show sensitivity to the Distinct Parts principle. If, however, conceptual individuation is a product of the linguistic encoding of count syntax or telicity of predicates, English speakers should show sensitivity to the Distinct Parts principle exclusively or more strongly than Mandarin speakers. In Experiment 2, we test the Distinct Parts principle with adult English and Mandarin speakers, again using the experimental stimuli and paradigm from Lee et al. (2024).

### 4.1 Experiment 2a: Objects

#### 4.1.1 Participants

Forty‐eight new adult native speakers of English and 47 new adult native speakers of Mandarin Chinese participated.

#### 4.1.2 Stimuli

Stimuli were adapted from Lee et al. (2024). Lee et al. (2024) used the 16 pairs of original object–substance images for target items used in Experiment 1a to extract two different 80 × 80 pixel parts from each image. One part was extracted from the center of each entity (middle part) and another part from the top right corner of each entity (edge part). See Table 5 for examples. The center and edge parts of each entity were selected to make the parts maximally distinct from each other, for both objects and substances. Moreover, the selection of center and edge parts of spatial entities was later mirrored in the selection of middle and end segments of temporal entities in Experiment 2b.

**Table 5:** Sample images (parts) in Experiment 2a

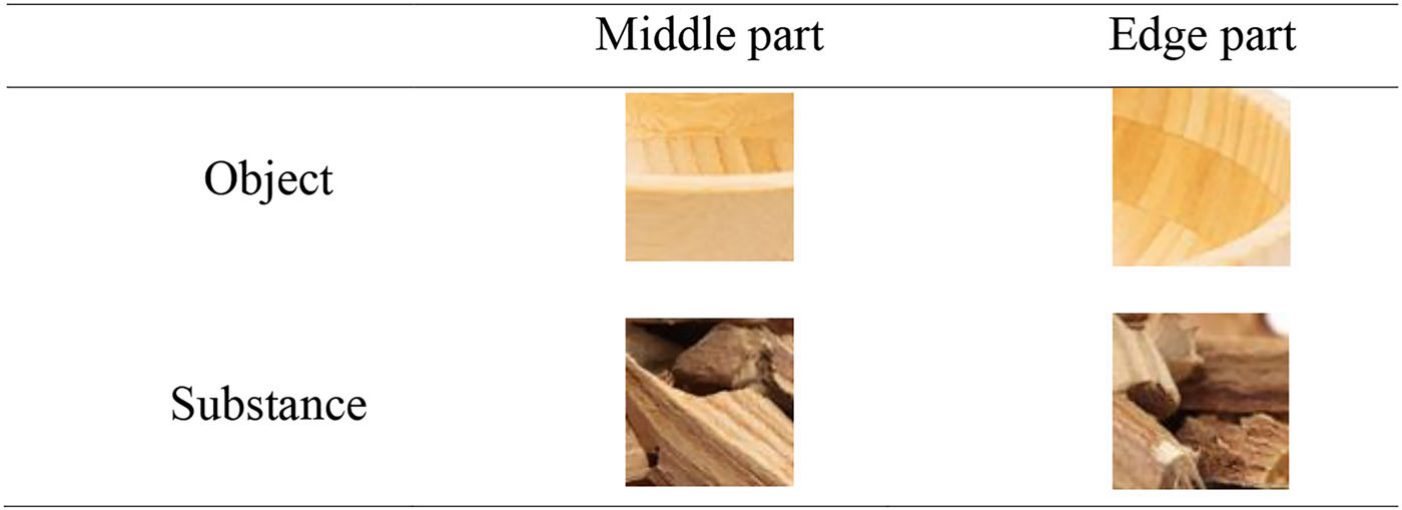

*Note*. Images in this table are representative of the stimuli used in the experiment but are not the actual stimuli due to copyright restrictions. The actual stimuli can be accessed in full on our OSF page.

We also included 12 filler items based on the fillers of Experiment 1a. Six of the filler items were identical image pairs. The other six of the filler items involved middle and edge parts of filler images (similar to the target items). Half of the changing and half of the unchanging fillers were taken from objects, and the other half from substances.

#### 4.1.3 Procedure

The trial structure of Experiment 2a was similar to that of Experiment 1a. At the beginning of each trial, a fixation cross was displayed for 1000 ms. After the fixation cross, one part of an entity was displayed for 100 ms. Then, the screen was masked as in Experiment 1a for 3000 ms. Afterward, the other part of the entity was displayed for 100 ms. There was no post‐mask after the other part of the entity. The ordering of the segments was counterbalanced so that in one half of the trials, participants saw the middle segment first, and in the other half, they saw the edge segment first. At the end of each trial, participants indicated whether the two images were identical or not by pressing a button (English: “Different or Identical?”; Mandarin: “剪辑过 (F) 还是相同的 (J)”).

#### 4.1.4 Results and discussion

**Fig. 3:**
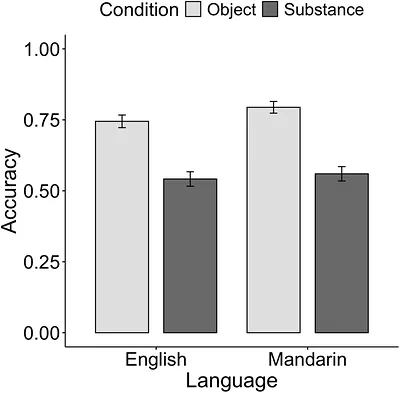
Mean accuracy by Language and Condition in Experiment 2a. (Error bars represent ± *SE*.)

Results from Experiment 2a are shown in Fig. 3. We coded Condition (Object = 0.5, Substance = −0.5) and Language (English = 0.5, Mandarin = −0.5) using centered contrasts and included them as fixed effects. Random effects included intercepts for participants and items, as well as by‐participant slopes for Condition and by‐item slopes for Condition, Language, and their interaction. Because the maximal model did not converge, the model reported here included intercepts for participants and items and by‐participant and by‐item random slopes for Condition. As expected by the Distinct Parts principle, there was an effect of Condition (*β* = 1.42, *SE* = 0.35, *z* = 4.06, *p* < .001): Participants were more likely to accurately identify the two segments as distinct for Objects (*M* = 0.77, *SD* = 0.42) than for Substances (*M* = 0.55, *SD* = 0.50). There was no effect of Language (*β* = −0.24, *SE* = 0.25, *z* = −0.97, *p* = .332) nor an interaction with Condition (*β* = −0.25, *SE* = 0.33, *z* = −0.75, *p* = .452).

Could low‐level visual properties of our stimuli have contributed to the accuracy difference between the Object and Substance conditions in Experiments 1a and 2a? To evaluate this possibility, we added a *z*‐scored pixel‐difference measure (between the original and restructured versions in Exp 1a and between the middle and the edge part in Exp 2a) as a fixed effect in the model. These analyses revealed that pixel differences did not affect accuracy in Experiment 1a (*β* = 0.29, *SE* = 0.17, *z* = 1.76, *p* = .079) nor Experiment 2a (*β* = 0.006, *SE* = 0.21, *z* = 0.03, *p* = .978). Furthermore, in both experiments, adding the pixel difference measure as a fixed effect did not change the significance of the Condition effect. These analyses confirm that the Object–Substance differences arise from abstract mental conceptualizations of individuation rather than lower‐level perceptual properties of our stimuli.

These results support the Distinct Parts principle, according to which individuals, such as objects, possess distinct parts. Both English and Mandarin speakers were sensitive to this principle in the object domain.

### 4.2 Experiment 2b: Events

#### 4.2.1 Participants

Forty‐eight new adult native speakers of English and 47 new adult native speakers of Mandarin Chinese participated.

#### 4.2.2 Stimuli

Following Lee et al. (2024), each original video from Experiment 1b was segmented into nine temporal segments in Experiment 2b. The fifth (middle) and the eighth (end) segments were used (see Table 6). This mirrors the stimuli design in Experiment 2a, where the middle and edge parts of spatial entities were selected. In addition to the target items, we included 12 filler items that were identical to the filler items used in Experiment 1b.

**Table 6:** Sample videos (segments) in Experiment 2b

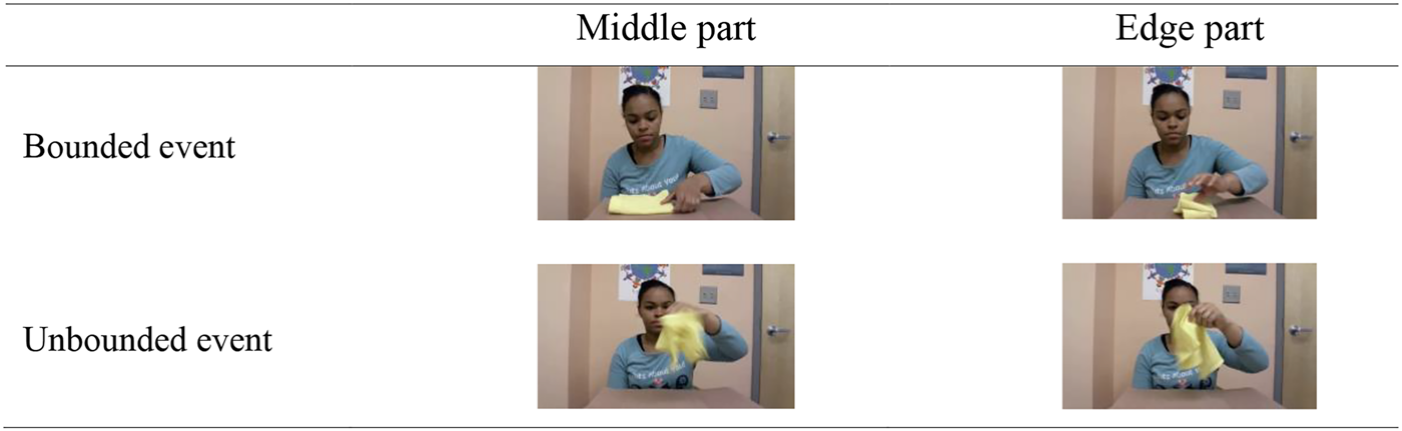

#### 4.2.3 Procedure

The trial structure of Experiment 2b was similar to that of Experiment 1b. At the beginning of each trial, a fixation cross was displayed for 1000 ms. Once the fixation cross disappeared, participants watched a video segment. Then, the screen was masked for 1500 ms. Afterward, participants watched the other video segment. The ordering of the segments was counterbalanced so that in half of the trials, participants saw the middle segment first, and in the other half, they saw the end segment first. At the end of each trial, participants used a button press to indicate whether they were identical or not (English: “Different or Identical?”; Mandarin: “剪辑过 (F) 还是相同的 (J)”).

#### 4.2.4 Results and discussion

**Fig. 4:**
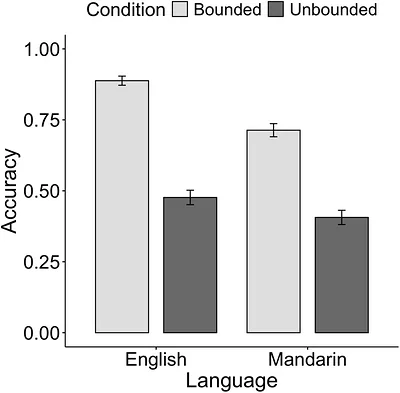
Mean accuracy by Language and Condition in Experiment 2b. (Error bars represent ± *SE*.)

Results are shown in Fig. 4. As in Experiment 1b, we entered Condition (Bounded = 0.5, Unbounded = ‐0.5) and Language (English = 0.5, Mandarin = −0.5) as fixed effects. The model reported here included intercepts for participants and items as random effects, as other models with more complex random effects structures failed to converge.

There was an effect of Condition: Participants were more likely to accurately identify the two segments as distinct for Bounded events than for Unbounded events (*β* = 2.48, *SE* = 0.17, *z* = 14.99, *p* < .001). There was also an effect of Language: English speakers were overall more accurate than Mandarin speakers (*β* = 0.98, *SE* = 0.28, *z* = 3.45, *p* < .001). Additionally, there was an interaction between Language and Condition (*β* = 0.93, *SE* = 0.30, *z* = 3.14, *p* < .01). To further understand this interaction, we analyzed each language group's accuracy data using generalized linear mixed effects models, with Condition (Bounded = 0.5, Unbounded = −0.5) as the fixed effect. Random effects started out with intercepts for participants and items, as well as by‐participant and by‐item slopes for Condition. The reported models included intercepts for participants and items as random effects and were the first models to converge. This analysis showed that English‐speaking participants were better at identifying the two segments as distinct for Bounded events than for Unbounded events (*β* = 2.98, *SE* = 0.26, *z* = 11.44, *p* < .001) and so were Mandarin‐speaking participants (*β* = 1.98, *SE* = 0.21, *z* = 9.42, *p* < .001). While both groups of speakers performed as expected by the Distinct Parts principle, this effect was stronger in English speakers.

What could explain this difference? One possibility is that this difference reflects a small but stable discontinuity in how speakers of English and Mandarin conceptualize individuals and associated properties, even outside of language. In other words, even though both language groups abide by the Distinct Parts principle of individuation in the event domain, English speakers are likely to draw a larger distinction between bounded and unbounded events than Mandarin speakers because of the crisper encoding of this distinction in the verbal system of English. As described earlier in detail, in English, verbs such as *fold* (telic) and *wave* (atelic) are specified for telicity, but this is not true in Mandarin. Similarly, in English, object noun phrases contribute to the calculation of telicity (e.g., *stick a sticker* vs. *stick stickers*), but in Mandarin, they often do not.

Another possibility, however, is that the observed difference between language groups in Experiment 2b is due to participants’ covert use of their native languages to encode the stimuli rather than by fundamental differences in non‐linguistic cognitive processes. Such online recruitment of language during the task would essentially suggest a language‐on‐language, as opposed to a language‐on‐thought, effect (see Gleitman & Papafragou, 2005, for this terminology; see also discussion of such effects in Section 1.2). Notice that, in Experiment 2b, participants were provided with short fragments of event stimuli and might have relied at least in part on linguistic recoding of these incomplete stimuli to interpret, remember, and/or compare them. If so, in English, using a telic description for a video may have encouraged an individuated (bounded) construal, and vice versa for an atelic description. But in Mandarin, the fact that bounded and unbounded events are often both likely to be encoded with atelic descriptions may have decreased the difference between the two conditions. Such online use of language would be transient and task‐dependent, not a robust feature of event conceptualization cross‐linguistically. We directly adjudicate between these two possibilities in Experiment 2c.

### 4.3 Experiment 2c: Events under verbal interference

In Experiment 2c, we directly test the nature of the role of language in Experiment 2b by replicating the experiment but using an additional verbal interference task. In these tasks, participants are required to repeat irrelevant linguistic material while simultaneously completing the main non‐linguistic task. This approach assumes that repeating the linguistic material occupies the phonological loop, thereby blocking inner speech, which might otherwise operate alongside (and affect) the nonverbal task (e.g., Trueswell & Papafragou, 2010). Previous studies in domains as disparate as motion or color perception have shown that cross‐linguistic differences in cognitive performance often disappear when verbal interference is introduced, a result widely interpreted to point to online (and thus malleable) involvement of language in those phenomena (Montero‐Melis & Bylund, 2017; Trueswell & Papafragou, 2010; Winawer et al., 2007; cf., Cardini, 2010; Feinmann, 2020; Lupyan, 2009).

If online recruitment of language was a factor in the degree of sensitivity to the Distinct Parts principle in Experiment 2b, verbal interference should eliminate the difference between the two language groups because covert linguistic encoding of the stimuli would no longer be possible. However, if English and Mandarin speakers conceptualize event individuation differently even outside of language, the difference across groups should be immune to verbal interference.

#### 4.3.1 Participants

Forty‐eight new adult native speakers of English and 46 new adult native speakers of Mandarin Chinese participated.

#### 4.3.2 Stimuli

The stimuli were identical to Experiment 2b.

#### 4.3.3 Procedure

The procedure was identical to Experiment 2b with one exception. At the beginning of every trial, participants were shown a 4‐digit sequence (e.g., 4935) for 5000 ms. At the beginning of the experiment, they were instructed to keep repeating the digits out loud during the main event task (following the linguistic interference task used in Trueswell & Papafragou, 2010). Participants were reminded at every trial to keep repeating the digits out loud. At the end of the trial (after the event task), participants were asked to write the 4‐digit sequence that they had seen at the beginning of the trial. The digits that participants had to repeat were different for each trial. This interference task served to suppress any linguistic encoding of events.

#### 4.3.4 Results and discussion

We planned to exclude participants whose accuracy on digit recall was below 75%. However, none of the participants’ accuracy fell below the 75% threshold (Mean accuracy = 0.97, *sd* = 0.18).

**Fig. 5:**
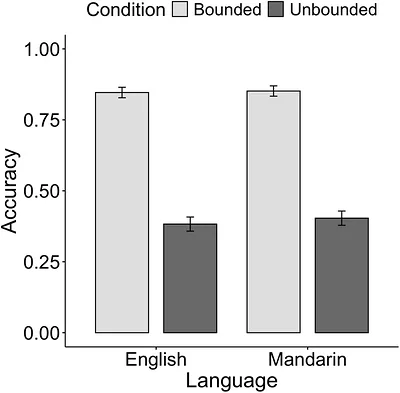
Mean accuracy by Language and Condition in Experiment 2c. (Error bars represent ± *SE*.)

Results are shown in Fig. 5. As in Experiment 2b, we entered Condition (Bounded = 0.5, Unbounded = −0.5) and Language (English = 0.5, Mandarin = −0.5) as fixed effects. As random effects, we included intercepts for participants and items, as well as by‐participant random slopes for Condition and by‐item random slopes for Condition and Language. The model reported here was the first model to converge and included intercepts for participants and items as random effects.

There was an effect of Condition: Participants were more likely to accurately identify the two segments as distinct for Bounded events than for Unbounded events (*β* = 2.79, *SE* = 0.16, *z* = 17.43, *p* < .001). There was no effect of Language (*β* = −0.08, *SE* = 0.21, *z* = −0.37, *p* = .71) nor a Condition by Language interaction (*β* = 0.02, *SE* = 0.28, *z* = 0.08, *p* = .935). Under verbal interference, the cross‐linguistic difference in Experiment 2b disappeared.

This last conclusion was confirmed in a generalized linear mixed effects model analysis with Condition (Bounded = 0.5, Unbounded = −0.5), Language (English = 0.5, Mandarin = −0.5), and Experiment (Exp.2b/No Verbal Interference = 0.5, Exp.2c/Verbal Interference = −0.5) as fixed effects (and intercepts for participants and items, as well as by‐participant random slopes for Condition and by‐item random slopes for Condition, Language, Experiment, and their interactions). We report the model including by‐participant and by‐item intercepts, as more complex models failed to converge. We found a three‐way interaction between Condition, Language, and Experiment (*β* = 0.91, *SE* = 0.41, *z* = 2.23, *p* = .026): the interaction between Condition and Language was modulated by the presence of the verbal interference task. Additionally, there was an effect of Condition (*β* = 2.62, *SE* = 0.11, *z* = 23.02, *p* < .001), an effect of Language (*β* = 0.43, *SE* = 0.17, *z* = 2.47, *p* = .013), a Condition × Language interaction (*β* = 0.46, *SE* = 0.20, *z* = 2.24, *p* = .025), a Condition × Experiment interaction (*β* = −0.49, *SE* = 0.20, *z* = −2.39, *p* = .017), and a Language x Experiment interaction (*β* = 0.01, *SE* = 0.35, *z* = 2.90, *p* < .01). There was no effect of Experiment (*β* = −0.01, *SE* = 0.17, *z* = −0.07, *p* = .94).

Results from Experiment 2c show that as in Experiment 2b, both English and Mandarin speakers were sensitive to the Distinct Parts principle in the event domain (Lee et al., 2024). In addition, a subtle but reliable cross‐linguistic difference in Experiment 2b did not survive when linguistic encoding was suppressed through a verbal interference task (Experiment 2c). Based on these results, we conclude that optional and flexible recruitment of language drove the cross‐linguistic differences in Experiment 2b. In that sense, this effect is reminiscent of similar, significant but transient effects of language in tasks where participants had the option to engage in linguistic encoding of the stimuli (Montero‐Melis & Bylund, 2017; Trueswell & Papafragou, 2010; Winawer et al., 2007; among others).^7^

Currently, evidence for the online involvement of language in our data remains limited: Unlike Experiment 2b, these online effects did not emerge in the object counterpart of this paradigm (Experiment 2a) and were also absent in Experiments 1a and 1b. Previous studies have suggested that linguistic recoding of stimuli happens in more demanding tasks where people use language as an additional cognitive tool (e.g., Trueswell & Papafragou, 2010). As alluded to already, it is possible that, in Experiment 2b, viewers might have relied at least in part on linguistic recoding of the partial event stimuli in the perceptual input to help resolve the uncertainty in terms of what the stimuli depicted, as well as remember and/or compare them. We conclude that, while online effects of linguistic influence can manifest in certain situations, they may not be as consistent as core effects of non‐linguistic individuation. In fact, results from all three experiments (Experiments 2a–c) showed cross‐linguistic sensitivity to a signature of individuation (Distinct Parts principle) that crosscuts the object and event domain.^8^

## 5 General Discussion

### 5.1 Signatures of individuation across domains and languages

In this study, we investigated whether conceptual considerations of individuation are language‐independent. Results from previous studies (Lee et al., 2024) have uncovered object–event correspondences in cognition; however, these findings came from speakers of English, a language that has robust means of encoding individuation in both the nominal and verbal domains (count syntax and telicity of predicates). Are cross‐domain considerations of individuation shaped through language? Here, we tested speakers of English and Mandarin, based on cross‐linguistic differences in the expression of individuation in nominal and verbal domains. English uses count–mass syntax and telicity (in monomorphemic verbs and their nominal arguments) to mark linguistic individuals in the nominal and verbal domains, respectively, and Mandarin lacks these overt linguistic features. Specifically, we tested whether conceptual principles of individuation along the lines of the No Structuring and Distinct Parts principles (Lee et al., 2024) are universally shared, regardless of the way individuation is expressed in one's language.

In Experiment 1, both English and Mandarin speakers were sensitive to the No Restructuring principle across both objects and events, according to which individuated entities resist structural restructuring. In Experiment 2, both English and Mandarin speakers were sensitive to the Distinct Parts principle, again across both objects and events, according to which individuated entities possess distinct parts. Experiment 2 also revealed that while the sensitivity is shared across both groups, English speakers had a stronger sensitivity to the Distinct Parts principle in the event domain (Experiment 2b). In order to examine whether these differences were driven by fundamental conceptual differences across language groups, we added a verbal interference task to suppress linguistic encoding of stimuli (Experiment 2c). When verbal interference was introduced, English and Mandarin speakers were equally sensitive to the Distinct Parts principle; that is, the cross‐linguistic differences disappeared. These findings converge with work from other domains, where cross‐linguistic differences on a non‐linguistic task were eliminated in the context of a verbal interference task (Trueswell & Papafragou, 2010; Winawer et al., 2007). Our results are compatible with the idea that while language affects performance on non‐linguistic tasks, it may not fundamentally alter the underlying cognitive systems.

Taken together, these results throw light onto the nature of entity categories in the human mind: Both English‐speaking and Mandarin‐speaking viewers process individuated and non‐individuated entities differently, with only the former having a well‐defined (temporal/spatial) structure with integrally ordered, distinct parts. These features of non‐linguistic individuation are conceptualized in similar ways cross‐linguistically and are potentially universal. These results, combined with earlier findings on non‐linguistic object–event correspondences in English speakers (e.g., Lee et al., 2024; Papafragou & Ji, 2023; see Kuhn et al., 2021; Wellwood et al., 2018, for findings using linguistic tasks), highlight the robustness of the object–event correspondence across languages. Furthermore, our approach offers a method for exploring this correspondence in a wider sample of diverse languages known to differ in terms of telicity (see Section 1.2).

### 5.2 Origins of individuation in the mind

Our findings support the idea that conceptual individuation is a product of the human mind prior to (and independently from) language. In further support of this idea in the spatial domain, research on preverbal infants indicates that infants younger than 1 year can differentiate between objects and substances: Infants, like adults, show impaired quantification and tracking abilities for substances relative to objects, and thus react differently to entities from each category (e.g., Huntley‐Fenner et al., 2002; vanMarle & Scholl, 2003; vanMarle & Wynn, 2011; see Rips & Hespos, 2015, for a review). This suggests that the fundamental ontological distinction between objects and substances emerges prior to language acquisition (Landau, Smith, & Jones, 1992; Soja, 1992; Soja et al., 1991, 1992). In a separate literature on events, there is evidence that young children can access the boundedness distinction when processing visual events in a non‐linguistic task (Ji & Papafragou, 2024).

Findings from our study raise the possibility that these early non‐linguistic distinctions between object–substance and bounded–unbounded events are rooted in a more abstract, domain‐general distinction between individuals and non‐individuals. Under this position, the representation of basic individuals (mental units)—a more deep‐seated and general representation than physical objects—may be part of core cognition. In fact, these domain‐general conceptions of mental units extend beyond the physical and visual world. For example, our mind identifies and interacts with units of auditory experience like a chirp, a beep, and a swoosh and units of internal experience such as an acute pain, a worry, and a gut feeling. At a more complex level, abstract entities such as protocols, networks, and governments are also individuated units with intricate internal structure. Despite their differences, a chirp and a government are alike at some level of human cognition in that they are both individuals. Domain‐general conceptual considerations that span across chirps and governments transcend domain‐specific cues such as object shapes and event boundaries, offering a broader framework for understanding core cognition. Ultimately, these considerations allow us to interpret and navigate the rich and multifaceted nature of human experience.

In addition to the current study, which suggests a potentially universal conception of individuals across cultures, future evidence indicating that considerations supporting mental units (No Restructuring, Distinct Parts, No Breaks) are available to preverbal infants would be able to provide further support for this position. These potentially preverbal and universal conceptual representations may in turn underpin and structure the linguistic encoding of individuation. Furthermore, early sensitivity to these representations may be used to map entity concepts onto foundational semantics in natural language during language development. Then, observations about natural language can in turn reveal core conceptual distinctions that characterize non‐linguistic thought.

### 5.3 Individuation in language and thought

Finally, our study contributes to the debate on the extent to which human cognition is susceptible to linguistic effects (for discussion, see Choi & Bowerman, 1991; Gleitman & Papafragou, 2005; Landau, 2022; also reviews in Bowerman & Levinson, 2001; Gentner & Goldin‐Meadow, 2003; Wolf & Holmes, 2011). We conclude that foundational aspects of object and event cognition, such as the conceptual features of basic units in both domains, are cross‐linguistically shared and potentially universal in human cognition, regardless of the specifics of how they are encoded in one's language.

Nevertheless, our studies also show that cross‐linguistic differences can be observed when viewers have online access to inner language. In particular, having clear linguistic means to distinguish between categories (e.g., bounded vs. unbounded events) can potentially aid tasks that require such a distinction in the cognitive domain, even when language is not overtly required. Our results also suggest that these linguistic effects are context‐sensitive. Our studies were not designed to identify the exact conditions under which these linguistic effects appear nor the time course of these effects. Future work could further examine the extent to which linguistic encoding happens in object and event perception and the exact circumstances under which language is recruited in a way that affects performance on cognitive tasks. Such work could also extend the languages sampled, including comparisons to sign languages. Finally, our results are compatible with the view that language offers an important cognitive tool that can optionally help cognitive tasks but does not fundamentally alter underlying cognitive representations (Gleitman & Papafragou, 2005; Trueswell & Papafragou, 2010; Landau et al., 2010).

## Supporting information

Supporting Information

## Data Availability

All stimulus materials, trial‐level data, and analysis code are available on the OSF page (https://osf.io/k487a).

## Notes

1 Lee et al. (2024) also demonstrated the effects of a third principle, the “No Breaks” principle, according to which individuated entities resist breaks (i.e., interruptions). This principle is not discussed further here for space reasons.

2 There are exceptions. For example, count nouns can appear in their bare form when they are interpreted as mass nouns via a “universal grinder” operation (e.g., *After the accident there was dog all over the road*). Conversely, mass nouns can be treated as count nouns via a “universal packager” operation (e.g., *Three beers please*; Jackendoff, 1991; Pelletier, 1975).

3 Telicity relates to the inherent temporal properties of verbs and predicates and thus represents what is often referred to as *lexical aspect* (to be distinguished from *grammatical aspect*, such as the perfective/imperfective distinction; Madden & Ferretti, 2009).

4 For completeness, we note that aspectual markers such as ‐*le, ‐guo, ‐zhe*, and *‐zai* and word order are also relevant for telicity in Mandarin, even though not the focus here (see Li & Thompson, 1981; Smith, 1991; Mangione & Li, 1993; Klein et al., 2000).

5 While numeral‐classifier phrases (e.g., *sān běn shū* “three CL book”) can delimit events in the same way as English quantized noun phrases, such phrases are less preferred than bare nouns unless it is necessary to specify the quantity of the referent.

6 The masking duration was shorter for events than spatial entities to offset the length of the event stimuli.

7 Naturally, in everyday situations, language is typically used without the inhibitory strategies employed in these experimental settings. We therefore interpret the results of our own and other interference tasks in this literature as showing that the human mind can adapt to using different strategies in various contexts. Our results suggest that language can sometimes, but not always, affect cognitive processes; in addition, the mental strategies used in a cognitive task can change when language is not available (for a similar perspective, see Ünal & Papafragou, 2016).

8 Recall that Mandarin‐speaking participants resided in various parts of the world including non‐Mandarin‐speaking countries. According to their language background reports, 98% of them had been born in a Mandarin‐speaking region and had lived there for an average of 20.60 years (note that their average age was 33.39 years). Furthermore, their average self‐reported proficiency in English was 5.73 out of 10. One might wonder whether these participants’ performance was affected by their current environment and/or level of proficiency in English. To address this possibility, we conducted an analysis on responses from Mandarin‐speaking participants collapsing across experiments using multiple linear regression with length of residence abroad and self‐reported English proficiency as predictors. (Both predictors were entered simultaneously into the model.) Our measure was the accuracy difference between the Object and Substance conditions (in Experiments 1a and 2a) and the Bounded and Unbounded event conditions (in Experiments 1b, 2b, and 2c). We found that neither length of residence abroad nor (self‐reported) English proficiency predicted Mandarin speakers’ performance (*p* = .24 and *p* = .90 respectively; similar results emerged when object and event tasks were analyzed separately).
