# Supplementary material for: Shared Principles of Individuation Underpin Object–Event Correspondences Cross‐Linguistically
Source: Cogn Sci. 2026 Aug 19;50(8):e70248. doi: 10.1111/cogs.70248 (PMC13490629; doi:10.1111/cogs.70248)
Supplement: Supplementary file 1 [file COGS-50-e70248-s001.docx]

**Supplementary Materials**

In addition to the frequentist analyses reported in the paper, we conducted supplemental Bayesian analyses for all experiments to address potential concerns about a Type II error for the interaction effects. Results of the Bayesian analyses indicated the same set of findings as our frequentist analyses. Bayesian models were estimated using the brms package (Bürkner, 2017) in R (R Core Team, 2024). For these analyses, we used weakly informative priors that incorporate knowledge about the baseline accuracy and condition effect from a previous set of experiments using the same stimuli, task, and design (Lee et al., 2024), while remaining intentionally conservative to avoid over-constraining the posterior. The analysis script used for these analyses can be found on <https://osf.io/k487a/?view_only=3b9110bfbb3e4f939bf939c6f38c9c18>.

**A1. Experiment 1**

**A1.1 Experiment 1a**

For the intercept, we placed a Normal(1.38, 0.50) prior. The mean corresponds to the log-odds of the overall accuracy observed in the prior study (Lee et al., 2024, Experiment 1a), averaged across conditions. For the condition effect, we used a Normal(1.0, 1.0) prior. The previous experiment reported a large effect of Object versus Substance, but because effect sizes can vary across samples, we adopted a skeptical informative prior, reflecting the expectation of a positive and potentially sizable difference, while the large standard deviation (1.0) permits considerable uncertainty. For random-effects standard deviations (participants and items), we used weakly informative Student-t(3, 0, 2.5) priors. Together, these priors express reasonable expectations grounded in empirical evidence while ensuring that the data from the present experiment remain the primary driver of posterior estimates. To evaluate the possibility that low-level visual properties of our stimuli have contributed to accuracy, we added a z-scored pixel‐difference measure (between the original and restructured versions) as a fixed effect in the model.

The model output indicated that accuracy was substantially higher in the Object condition than in the Substance condition (β = -2.14, 95% CrI [-2.86, -1.36]). In contrast, we found no evidence for a language difference (β = 0.26, 95% CrI [-0.31, 0.84]) and no reliable evidence that this effect differed across language groups, i.e. no interaction between condition and language (β = 0.05, 95% CrI [-0.85, 0.96]). Pixel difference did not reliably predict accuracy (β = 0.25, 95% CrI [-0.11, 0.62]). This indicates that the magnitude of the condition effect was consistent across both language groups. Furthermore, the accuracy difference between Object and Substance conditions is not explained by differences in pixel‐level visual change.

**A1.2 Experiment 1b**

As in Experiment 1a, priors were informed by a previous experiment using the same design and task (Lee et al., 2024, Experiment 1b). The intercept prior (Normal(1.19, 0.50)) corresponds to the mean accuracy observed previously, with wide uncertainty. The condition effect received a skeptical Normal(0.50, 1.0) prior based on the prior Bounded-Unbounded difference. We placed weakly informative Normal(0, 1) priors on the Language effect and interaction, as no prior evidence was available for these terms. Random-effects standard deviations used weakly informative Student-t(3, 0, 2.5) priors.

Accuracy was higher in the Bounded condition than in the Unbounded condition (β = -1.00, 95% CrI [-1.90, -0.04]). We found no evidence for a language difference (β = 0.23, 95% CrI [-0.34, 0.81]) and no evidence for an interaction between condition and language (β = -0.11, 95% CrI [-0.90, 0.70]). Thus, while the Bounded–Unbounded contrast remained present, its magnitude did not differ reliably across language groups.

**A2. Experiment 2**

**A2.1 Experiment 2.1**

Priors were weakly informative and guided by a previous experiment using the same task and stimuli (Lee et al., 2024, Experiment 2a). To test whether low-level visual differences between the two images in each item could explain performance, z-scored mean absolute pixel-difference measure was included as a covariate. The intercept was given a Normal(0.87, 0.50) prior, corresponding to an expected overall accuracy of roughly 70% on the logit scale with substantial uncertainty. Because the earlier study found higher accuracy for objects than substances, the condition effect received an informed but moderately wide Normal(1.1, 1.0) prior, allowing for effects of similar magnitude while accommodating considerable variability. No prior expectations were available for Language differences, the interaction between Condition and Language, or Pixel differences, so they were assigned weakly informative Normal(0, 1) priors. Standard weakly regularizing Student-t(3, 0, 2.5) priors were used for all random-effects standard deviations.

Accuracy was substantially higher in the Object condition than in the Substance condition (β = 1.37, 95% CrI [0.59, 2.15]). There was no evidence for a difference between language groups (β = -0.22, 95% CrI [-0.71, 0.27]), nor any indication that the Condition effect varied by Language (β = -0.24, 95% CrI [-0.90, 0.44]). Thus, the robust Object–Substance advantage was comparable across language groups. Pixel differences themselves did not predict accuracy (β = 0.04, 95% CrI [-0.41, 0.48]).

**A2.2 Experiment 2b**

Priors were weakly informative and guided by a previous experiment using the same task and stimuli (Lee et al., 2024, Experiment 2b). The intercept was assigned a Normal(0.72, 0.50) prior, reflecting an expected overall accuracy of roughly 67% on the logit scale with substantial uncertainty. Because the earlier experiment showed markedly higher accuracy for bounded than unbounded events, the Condition effect received an informed Normal(2.22, 1.0) prior, allowing for effects of similar magnitude while remaining conservative. No prior expectations were available for language differences or the interaction, so both were given weakly informative Normal(0, 1) priors. Random-effects standard deviations were assigned regularizing Student-t(3, 0, 2.5) priors.

Accuracy was considerably higher for bounded events than for unbounded events (β = 2.55, 95% CrI [1.48, 3.60]). We also observed a language difference, with English speakers showing overall higher accuracy than Mandarin speakers (β = 1.05, 95% CrI [0.39, 1.72]). Additionally, the interaction between Condition and Language showed a positive trend (β = 0.84, 95% CrI [-0.02, 1.70]), suggesting that the Bounded-Unbounded difference may be somewhat larger for the English-speaking group, although the credible interval included zero.

**A2.3 Experiment 2c**

We used the same set of priors as Experiment 2b. Accuracy was substantially higher for bounded than for unbounded events (β = 2.81, 95% CrI [1.71, 3.86]). There was no evidence for a language difference in overall accuracy (β = -0.10, 95% CrI [-0.68, 0.48]) and no indication of an interaction between condition and language (β = 0.08, 95% CrI [-0.62, 0.78]). Thus, in this experiment the Bounded–Unbounded contrast was large and robust across languages, with no evidence that language modulated the size or direction of the effect.
